# The Damage Effects on a HgCdTe Detector of a Short-Infrared Pulsed Laser with Different Pulse Widths

**DOI:** 10.3390/mi17070813

**Published:** 2026-07-06

**Authors:** Qiheng Wei, Xianfeng Wu, Lingyuan Wu, Yongqiang Zhang, Fuli Tan, Bo Fu, Wei Li, Yanglong Li

**Affiliations:** Institute of Fluid Physics, China Academy of Engineering Physics, Mianyang 621900, China; weiqiheng@caep.cn (Q.W.); wxftxdy@163.com (X.W.); wuly@caep.cn (L.W.); zhangyongqiang@caep.cn (Y.Z.); tanfuli2008@163.com (F.T.); fubo@caep.cn (B.F.)

**Keywords:** laser irradiation effect, HgCdTe, short infrared, pulse width

## Abstract

The high sensitivity of HgCdTe infrared detectors makes them highly vulnerable to laser irradiation, yet the influence of pulse width on damage behavior in the short-wave infrared (SWIR) band remains insufficiently understood. In this study, we experimentally and numerically investigate the damage effects of SWIR pulsed lasers on HgCdTe focal plane array detectors, focusing on the role of pulse width. Three lasers with pulse widths of 5.5 ns, 0.6 ms and 2 ms are used to irradiate the detector, and the damage thresholds for spot damage, line damage, and complete failure are measured. Damage morphologies are characterized by optical microscopy and scanning electron microscopy. A finite-element thermal model is also established to calculate transient temperature distributions and theoretical damage thresholds. For the 0.6 ms pulse, the measured thresholds for spot damage, line damage, and complete failure are 5.7 J/cm^2^, 65.4 J/cm^2^, and 157.3 J/cm^2^, respectively; for the 2 ms pulse, these increase to 12.1 J/cm^2^, 149.3 J/cm^2^, and 405 J/cm^2^ due to energy dispersion. Microscopic analysis reveals that spot damage arises from melting of HgCdTe and indium bumps, line damage from partial damage to the read-out integrated circuit (ROIC) layer, and complete failure from melt-through of the ROIC layer. The spot damage threshold of the 5.5 ns pulse is 1.2 J/cm^2^, while neither line damage nor complete failure occurs even with a 352.5 J/cm^2^ laser pulse, indicating different damage mechanisms due to a thermal confinement effect. The simulation results agree well with the experimental observations. These findings clarify the pulse-width dependence of damage thresholds and provide practical guidance for detector hardening and photoelectric countermeasure design.

## 1. Introduction

HgCdTe (mercury cadmium telluride) is a direct-bandgap semiconductor that has become the dominant material for infrared detection owing to its tunable bandgap, high quantum efficiency, and fast response [1,2]. By adjusting the Cd composition, HgCdTe detectors can cover the entire infrared spectrum, including the short-wave infrared (SWIR, 1–3 µm), mid-wave infrared (MWIR, 3–5 µm), and long-wave infrared (LWIR, 8–14 µm) atmospheric windows [3,4]. These detectors are widely used in space exploration, target tracking, and environmental monitoring [5,6]. However, the high sensitivity that makes HgCdTe so valuable also renders it extremely vulnerable to laser irradiation. With the rapid development of high-energy lasers, HgCdTe detectors are increasingly threatened by laser dazzling and even irreversible damage, which can severely degrade or completely disable the imaging system [7,8].

Over the past five decades, extensive research has been conducted on laser-induced damage in HgCdTe materials and detectors. Bartoli and his team pioneered the theoretical understanding of thermal damage mechanisms in HgCdTe photoconductive and photovoltaic detectors under both pulsed and continuous-wave laser irradiation [9,10,11,12]. They developed one-dimensional thermal models that revealed the crucial role of bonding layers as thermal bottlenecks, and they identified two distinct thermal recovery timescales associated with heat flow through epoxy and varnish layers.

Subsequent experimental studies have further explored the damage phenomena in HgCdTe detectors under various laser conditions. For HgCdTe detectors irradiated by LWIR lasers, researchers have observed shadow formation due to interfacial gaps between the photosensitive layer and the CdZnTe substrate, crack propagation initiated from the photosensitive layer, and the occurrence of dark lines caused by Hg precipitation that short-circuits the readout circuit [13,14]. In the mid-infrared band, Liu et al. [15] and Zhang et al. [16] investigated the damage thresholds of HgCdTe focal plane array (FPA) detectors and found that point damage, line damage, and complete failure occur sequentially with increasing laser energy density. Their three-dimensional finite-element simulations showed that thermal melting of the HgCdTe layer is the primary damage mechanism and that the HgCdTe layer reaches its melting point before the indium columns. In the near-infrared/SWIR region, Chen et al. [17] used a 10 ns Nd:YAG laser to irradiate HgCdTe crystals and measured a melting damage threshold of 8.4 × 10^6^ W/cm^2^, which agreed well with thermal model predictions. They also observed periodic ripple structures on the crystal surface and explained their formation using transverse surface acoustic wave theory. Li et al. [18] investigated the combined effect of continuous-wave (CW) and nanosecond lasers on CCDs, showing that a pre-irradiation CW laser can significantly reduce the damage threshold of the subsequent pulsed laser. Ma et al. [19] investigates the interference effects of a nanosecond pulsed laser and CW laser on PbS infrared detectors, a typical material for SWIR detection. The results show that at a CW laser fluence of 6.1 W/cm^2^, the PbS detector is unable to detect any signal, producing a temporary blind spot.

Despite these achievements, there remains a notable research gap regarding the influence of pulse width on the damage behavior of HgCdTe detectors in the SWIR band. Most previous studies have focused on either LWIR or MWIR wavelengths, and the pulse widths examined are often limited to specific values. Experimental and theoretical investigations applying different pulse widths in the SWIR band are still lacking.

In this paper, we present an experimental and simulation study on the damage effects of SWIR pulsed lasers on HgCdTe FPA detectors, with emphasis on the role of pulse width, in which a comparison of ultra-short-pulse and long-pulse damage mechanisms is provided. Three SWIR lasers with a pulse width of 5.5 ns, 0.6 ms and 2 ms are used to irradiate the detector, and the damage thresholds for point damage, line damage, and complete failure are measured. The corresponding damage morphologies are analyzed by optical microscope and scanning electron microscopy (SEM). Meanwhile, a finite-element thermal model is established. The model is used to calculate the transient temperature distributions, to determine the damage order of different layers, and to derive the theoretical damage thresholds for different pulse widths. The relationship between power density and pulse width is examined and compared with Bartoli’s classical scaling law. The results provide practical guidance for detector hardening and laser countermeasure design.

## 2. Materials and Methods

### 2.1. The Theoretical Model

To establish a theoretical model for the damage effects on a HgCdTe detector of a short-infrared pulsed laser, it is imperative to employ the heat conduction theory. For a homogeneous and isotropic material, the three-dimensional unsteady thermal conduction differential equation can be expressed as follows:

(1) $$\rho c\frac{\partial T}{\partial t}=k\nabla^{2}T+q,$$

where ρ is the density of the material, c is the specific heat capacity, T is the temperature, which is a function of spatial coordinates and time, and k is the thermal conductivity. q is the heat source term. For a HgCdTe detector, it is continuously cooled by a refrigeration system and maintained at 77 K. Thus, we can obtain the initial condition:

(2) $$T_{initial}=T_{0}.$$

Then employing the expression of the Laplace operator in Cylindrical Coordinates, Equation (1) becomes the following:

(3) $$\rho c\frac{\partial T}{\partial t}=k(\frac{1}{r}\frac{\partial }{\partial r}\left(r\frac{\partial T}{\partial r}\right)+\frac{1}{r^{2}}\frac{\partial^{2}T}{\partial \varphi^{2}}+\frac{\partial^{2}T}{\partial Z^{2}})+q.$$

The heat source term rises from the absorption of laser energy:

(4) $$q=\alpha I(1-R)e^{-\alpha z},$$

where α represents the absorption coefficient, R is the reflectivity, and I is the intensity of the laser. Considering that the laser beam has a Gaussian spot profile and the size of the material is much larger than the laser spot, we can get $\frac{\partial T}{\partial \varphi }=0$ due to the symmetry condition:

(5) $$\rho c\frac{\partial \theta }{\partial t}=k(\frac{1}{r}\frac{\partial }{\partial r}\left(r\frac{\partial \theta }{\partial r}\right)+\frac{\partial^{2}\theta }{\partial Z^{2}})+q.$$

where $\theta =T-T_{0}$ represents the temperature increase. Assuming the thermal damage occurs when the temperature reaches the melting point, we can get the damage threshold E_0_ [11], expressed as follows:

(6) $$E_{0}=E_{∆T}[1+\frac{\sigma \tau \alpha \pi^{1/2}}{w_{0}{tan}^{-1}({4\sigma \tau /{w_{0}}^{2})}^{1/2}}],$$

where $E_{∆T}=\frac{∆T_{th}\rho c}{(1-R)\alpha }$, τ represents the pulse width, $\sigma =\frac{k}{\rho c}$ is the thermal diffusivity, $w_{0}$ is the radius of the laser spot, and Δ*T*th denotes the temperature increase from initial state to the melting point.

### 2.2. Experimental Setup

**Figure 1:**
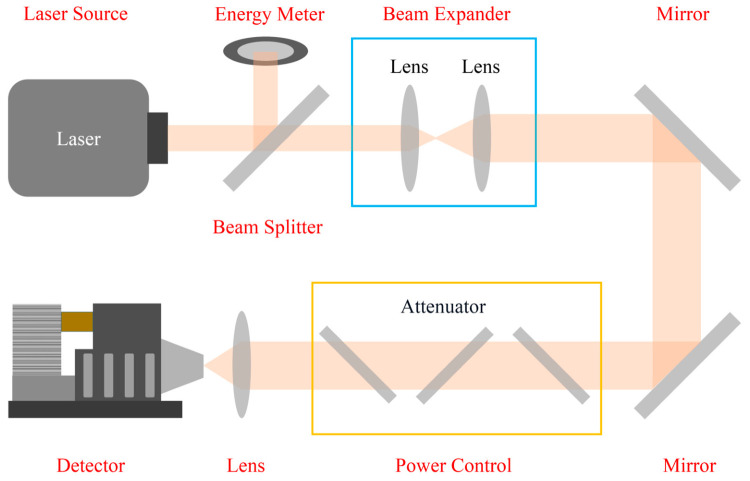
The experimental setup.

The experimental setup is shown in Figure 1. A short-infrared (2.7 μm) multimode pulsed laser beam is generated by a solid-state pulsed laser source, which is split into two parts by a beam splitter. One part of the beam, labeled monitoring beam, is used to monitor the pulse energy, and the other beam as the illumination beam applied to carry out the irradiation experiments. The max output energy for 5.5 ns, 0.6 ms and 2.0 ms is 0.5 J, 0.7 J, and 0.6 J per pulse, respectively. A beam expander that consists of two lenses is employed to expand the laser diameter to reduce the divergence angle. Reflected with two mirrors, the expanded laser beam enters the power control device, in which the pulse energy is modified with the reflective and absorptive attenuators. Then the beam is focused by a lens to irradiate the HgCdTe detector (Hg_1−x_Cd_x_Te, x = 0.3).

**Figure 2:**
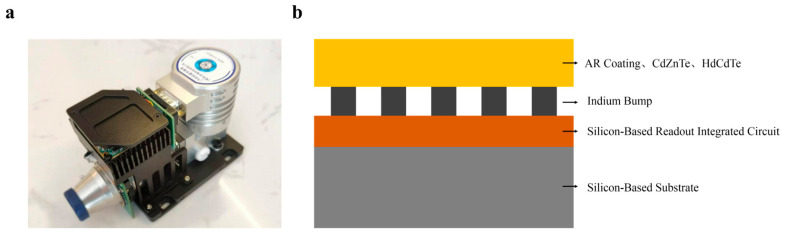
(**a**) An image of the detector; (**b**) a schematic diagram of the detector chip architecture.

The HgCdTe detector is a cooled infrared FPA Detector from the Abscience (Changzhou, China) Optoelectronic Technology Co., Ltd. (product model: AS-MW320P30), with a 30 μm × 30 μm pixel size and 320 × 256 plane array. The image of the detector and the schematic diagram of the detector chip architecture are shown in Figure 2. The detector chip is composed of four layers, from the top to the bottom, including the photosensitive layer, the indium bump layer, the ROIC layer, and the silicon-based substrate. The photosensitive layer consists of the ZnS anti-reflection (AR) coating, the CdZnTe substrate, and the HdCdTe. The indium bump layer consists of periodically arranged indium bumps and epoxy resin. The integration time is set to 3 ms and synchronized with the laser pulse.

## 3. Results and Discussion

### 3.1. The Experimental Results with a 0.6 ms Pulsed Laser

**Figure 3:**
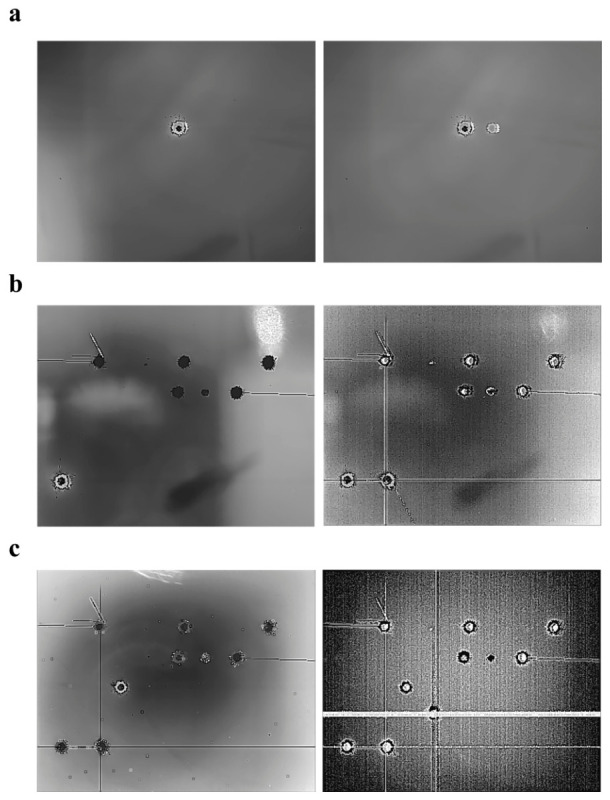
The experimental results of a 0.6 ms pulsed laser with a 2.7 μm wavelength. (**a**) Images of the detector before (**left**) and after (**right**) being irradiated by a 5.7 J/cm^2^ laser pulse; (**b**) images of the detector before (**left**) and after (**right**) being irradiated by a 65.4 J/cm^2^ laser pulse; (**c**) images of the detector before (**left**) and after (**right**) being irradiated by a 157.3 J/cm^2^ laser pulse.

The experimental results of a 0.6 ms pulsed laser with a 2.7 μm wavelength and 360 μm spot diameter are presented in Figure 3. In each experiment, a single pulse is employed to irradiate the detector at different positions. When the energy intensity reaches 5.7 J/cm^2^, spot damage occurs where several pixels lose the photosensitive ability in the laser spot center, as shown in Figure 3a. By increasing the power of the incident laser to 65.4 J/cm^2^, the phenomenon of line damage appears, in which all pixels along the line passing through the laser spot lose imaging capabilities, as presented in Figure 3b. Finally, with the irradiation of an energy intensity 157.3 J/cm^2^ laser pulse, the detector completely loses image ability, indicating complete failure. After irradiation, the detector outputs no signal, and the image is fixed and shows zero response to external light, as shown in Figure 3c.

**Figure 4:**
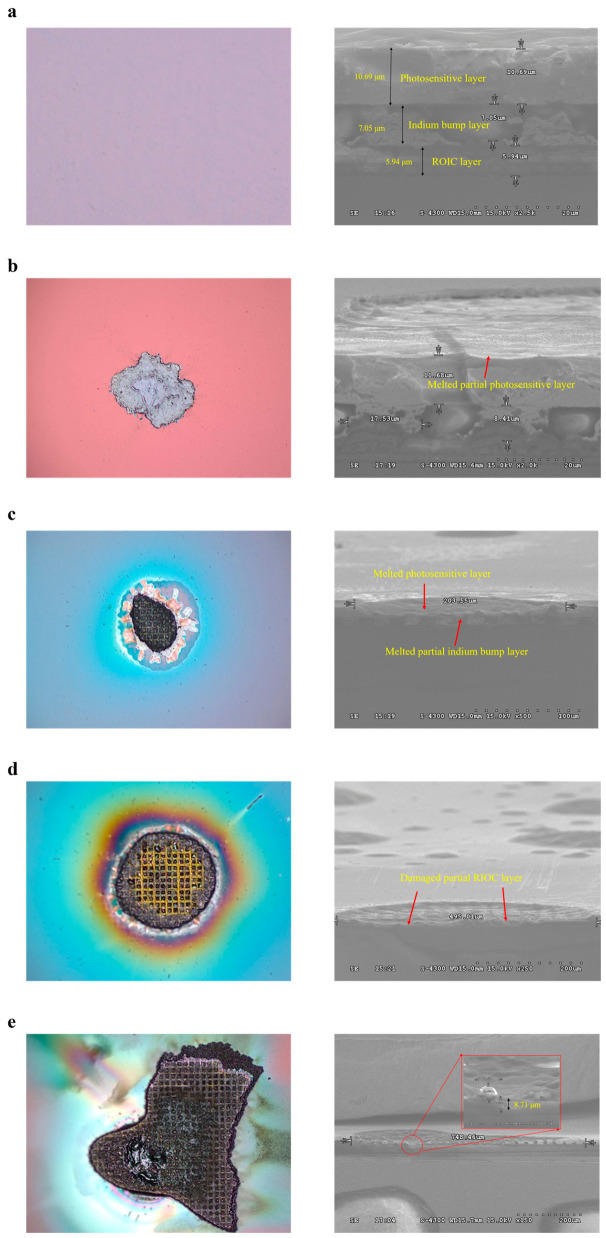
The optical microscope and SEM images of the chip. (**a**) Microscope (up) and SEM (down) images of the normal position; (**b**) microscope (up) and SEM (down) images of the spot damage area; (**c**) microscope (up) and SEM (down) images of the multiple-pixel damage region; (**d**) microscope (up) and SEM (down) images of the line damage area; (**e**) microscope (up) and SEM (down) images of the complete failure chip, where the image in the red square is the amplification of the partial image in the red circle. The depth below the indium bump layer is 8.71 μm, which is larger than the thickness of the normal RIOC layer, indicating that the ROIC layer is melted through.

To reveal the mechanism of the damage effect, we obtain optical microscope and SEM images to analyze the irradiated chip. Figure 4a shows the microscope and SEM images of the normal position, in which the microscope topography is smooth and even, and the SEM image indicates that the thickness of the photosensitive layer, the indium bump layer and the ROIC layer are about 10.69 μm, 7.05 μm, and 5.94 μm, respectively. Figure 4b presents the images of the spot damage area where the photosensitive layer is melted. The images of the multiple-pixel damage region are presented in Figure 4c. The results show that the photosensitive layer is melted through, and part of the indium bumps are damaged, resulting in a larger damaged region. Therefore, the mechanism of spot damage is melting of HgCdTe and indium bumps. To investigate the mechanism of line damage, we tested the optical and SEM image of the line damage area, as shown in Figure 4d. The results show that the photosensitive layer and part of the indium bump layer are melted through, and part of the ROIC layer is damaged, indicating that the mechanism of line damage is partial damage of the ROIC layer. By continuing to increase the energy intensity, the ROIC layer will be melted through, leading to complete failure of the detector, as shown in Figure 4e.

### 3.2. The Experimental Results with a 2 ms Pulsed Laser

**Figure 5:**
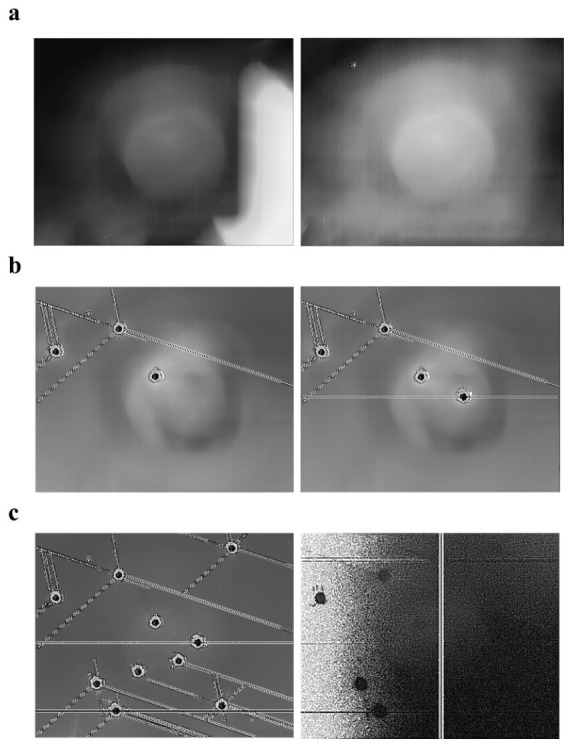
The experimental results of a 2 ms pulsed laser with 2.7 μm wavelength. (**a**) Images of detector before (**left**) and after (**right**) being irradiated by a 12.1 J/cm^2^ laser pulse; (**b**) images of detector before (**left**) and after (**right**) being irradiated by a 149.3 J/cm^2^ laser pulse; (**c**) images of detector before (**left**) and after (**right**) being irradiated by a 405 J/cm^2^ laser pulse.

To investigate the influence of pulse width in laser irradiation effects, we conducted an experiment where a 2 ms pulsed laser was employed to irradiate the HgCdTe detector, measuring the damage threshold. As shown in Figure 5 the thresholds of spot damage, line damage and complete failure are 12.1 J/cm^2^, 149.3 J/cm^2^, and 405 J/cm^2^, which are higher than those of the 0.6 ms pulsed laser due to energy dispersion.

### 3.3. The Experimental Results with a 5.5 ns Pulsed Laser

**Figure 6:**
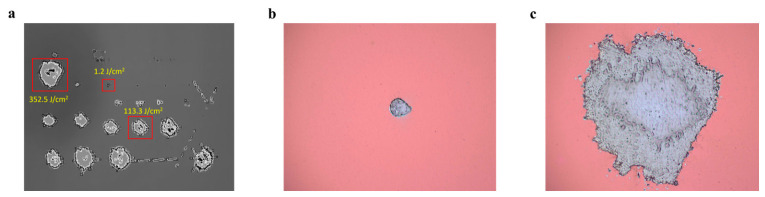
The experimental results of a 5.5 ns pulsed laser with a 2.7 μm wavelength. (**a**) An output image of detector after being irradiated by 1.2 J/cm^2^, 113.3 J/cm^2^, and 352.5 J/cm^2^ laser pulses; (**b**) microscope image of region irradiated by a 1.2 J/cm^2^ laser pulse; (**c**) microscope image of detector irradiated by a 352.5 J/cm^2^ laser pulse.

To investigate the influence of pulse width on laser irradiation effects, we conducted an experiment where a 5.5 ns pulsed laser was employed to irradiate the HgCdTe detector, measuring the damage threshold. As shown in Figure 6 the threshold of the spot damage is 1.2 J/cm^2^. However, even when the energy density reaches 352.5 J/cm^2^, neither line damage nor complete failure occurs. The optical microscope images show that the HgCdTe layer is not melted through with 352.5 J/cm^2^ laser irradiation, indicating different damage mechanisms.

To illuminate the phenomenon, we calculated the thermal diffusion length of 5.5 ns, 0.6 ms, and 2.0 ms pulsed lasers using the following equation:

(7) $$L=\sqrt{\frac{k\tau }{\rho c}}$$

where L is the thermal diffusion length. By employing the parameters in Table 1 and Table 2, we obtain the thermal diffusion lengths of 5.5 ns, 0.6 ms and 2 ms pulsed lasers. The results for 5.5 ns are $L_{HgCdTe}=0.22$ μm and $L_{In}=0.52$ μm. The results for 0.6 ms ns are $L_{HgCdTe}=73$ μm and $L_{In}=171$ μm. The results for 2.0 ms are $L_{HgCdTe}=134$ μm and $L_{In}=312$ μm. For the 0.6 ms and 2.0 ms pulsed laser, the thermal diffusion length is larger than the thickness of the HgCdTe and indium bump layer, indicating that the heat can completely penetrate the entire HgCdTe and indium layer during the laser pulse width and achieve line damage and complete failure. However, the thermal diffusion length of a 5.5 ns pulsed laser is less than 1 μm, making it hard to realize deep damage, such as line damage and complete failure. Therefore, this phenomenon can be explained by the thermal confinement effect. Moreover, according to the calculating results, the pulse duration of 0.6 ms results in a shorter action time, while under the 2 ms condition, thermal diffusion is more pronounced and covers a wider range. This dynamic difference in heat flux leads to the fact that when achieving the same damage state, the power density (energy density/pulse width) required for a 2 ms pulse width is lower than that for a 0.6 ms pulse width.

By employing Equation (6) and applying the physical parameters in Table 1, we can obtain the theoretical results of the spot damage threshold for the 5.5 ns, 0.6 ms and 2 ms pulsed laser as 1.2 J/cm^2^, 6.4 J/cm^2^ and 13.9 J/cm^2^, which are in good agreement with the experimental results.

### 3.4. The Finite-Element Simulation Model

According to the structure of the detector, a four-layer material model was created, consisting of a photosensitive layer, indium bump layer, ROIC layer, and silicon-based substrate from top to bottom, with thicknesses of 11 μm, 7 μm, 6 μm and 450 μm, respectively. The indium bump layer adopts a uniform structure for equivalent calculation, and the ablation temperature of the silicon-based ROIC is lower than that of the pure silicon substrate. The key thermophysical parameters of the materials used in the finite-element simulation model are shown in Table 2.

The distribution of the laser intensity follows a Gaussian distribution (the center of the light spot is at the origin), which can be expressed as follows:

(8) $$I=\frac{E_{ave}}{\eta \tau }exp(-\frac{2r^{2}}{{w_{0}}^{2}})\cdot pw(t),$$

where $E_{ave}$ is the average energy density of the pulse, and η  is the filling factor (the ratio of the average light intensity to the maximum light intensity) with a value of 0.432. The latter part, pw(t), is a rectangular wave function, which is used to realize pulse control (a single pulse is used in this study).

The simulation was carried out by the solid heat transfer module, and the material ablation removal process was realized by controlling the key parameters of the material through the piecewise function related to the ablation temperature. The phase transition process of materials was simulated by the apparent heat capacity method, and the expression for the phase change interval is given by $c=c_{1}+L_{m}/∆T_{m}$. Herein, $∆T_{m}$ is generally set to a range of 10~20 K. In order to simulate the heat transfer state when the upper material is ablated and the light source directly irradiates the exposed area of the lower layer, we define the anisotropic thermal conductivity in the area exceeding the ablation temperature, that is, by increasing the thermal conductivity in the incident direction and reducing the transverse thermal conductivity (approaching 0). Finally, the ablation region is obtained by time integration of the ablation velocity, and the ablation morphology is displayed through the “filter” setting. The ablation velocity can be expressed as follows:

(9) $$v_{n}=\frac{q_{a}}{\rho H_{s}}=\frac{h_{a}(T-T_{a})}{\rho H_{s}},$$

where $q_{a}$ represents the heat flux absorbed by the material ablation, $h_{a}$ represents the heat transfer coefficient, $T_{a}\;$ represents the ablation temperature, and $H_{s}\;$ represents the material ablation latent heat.

**Figure 7:**
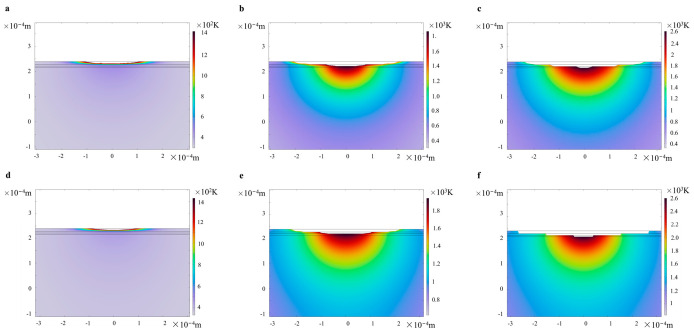
The simulation results. (**a**–**c**) The simulation results of the 0.6 ms pulsed laser with an energy density of 5.7 J/cm^2^, 65.4 J/cm^2^ and 157.3 J/cm^2^, respectively; (**d**–**f**) the simulation results of the 2 ms pulsed laser with an energy density of 12.1 J/cm^2^, 149.3 J/cm^2^ and 405 J/cm^2^, respectively.

Figure 7 shows three groups of the simulation results under the irradiation of 0.6 ms and 2 ms laser pulses. As shown in Figure 7a, a single 0.6 ms pulse with an energy density of 5.7 J/cm^2^ causes ablation on the photosensitive layer of the detector, which is consistent with the spot damage observed in the corresponding experiment. With an increase in energy density to 65.4 J/cm^2^, the ablation depth increases further. The indium bump layer is completely penetrated, and the transient temperature field starts to ablate the ROIC layer, as shown in Figure 7b, which corresponds to the linear damage observed experimentally. At an energy density of 157.3 J/cm^2^, the ROIC layer is perforated by ablation (Figure 7c), resulting in total failure of the detector and agreeing well with the damage severity recorded in the experiments.

Figure 7d–f show the simulation results for 2 ms pulses at energy densities of 12.1 J/cm^2^, 149.3 J/cm^2^ and 405 J/cm^2^, respectively. The ablation depths are similar to those in Figure 7a–c, and all results are consistent with the experimental results under the respective conditions.

## 4. Conclusions

In this work, we investigated the damage effects on a HgCdTe detector of a short-infrared pulsed laser and provided a comparison between ultra-short-pulse and long-pulse damage mechanisms. The experimental results show that the thresholds of spot damage, line damage, and complete failure are 5.7 J/cm^2^, 65.4 J/cm^2^, and 157.3 J/cm^2^ for the 0.6 ms pulse width laser, respectively. Meanwhile, with the larger pulse width of 2 ms, these thresholds increase to 12.1 J/cm^2^, 149.3 J/cm^2^, and 405 J/cm^2^ due to energy dispersion. By employing optical microscopy and SEM, we reveal that spot damage is induced by melting of HgCdTe and indium bumps, line damage arises from partial damage of the ROIC layer, and melting penetration of the ROIC layer leads to complete failure. For the 5.5 ns pulsed laser, the spot damage threshold is 1.2 J/cm^2^, while neither line damage nor complete failure occurs even with a 352.5 J/cm^2^ laser pulse, indicating different damage mechanisms due to the thermal confinement effect. Moreover, we established a theoretical model and a finite element simulation model, of which the results are consistent with experimental results. The findings presented are expected to provide substantial contributions to photoelectric countermeasure research.

## Figures and Tables

**Table 1:** The physical parameters used to derive the theoretical results [11].

Number	Parameter	Value
1	ρ	7 g/cm^3^
2	Δ*T*th	660 K
3	c	0.15 J/gK
4	α	10^3^ cm^−1^
5	R	0.05
6	σ	0.09 cm^2^/s
7	$w_{0}$	180 μm

**Table 2:** The physical parameters used to derive the theoretical results [11,20].

Physical Parameter	HgCdTe	Indium	Si
ρ (g/cm^3^)	7	7.3	2.33
c (J/gK)	0.15	0.23	0.7
k (Wcm^−1^K^−1^)	0.2	0.82	1.3
Melting temperature, $T_{m}\;$ (K)	993	430	1687
Latent heat of melting, $L_{m}$ (J/g)	130	28.5	1780

## Data Availability

The data presented in this study are available on request from the corresponding authors.

## Abbreviations

The following abbreviations are used in this manuscript:

SWIR	Short-Wave Infrared
MWIR	Mid-Wave Infrared
LWIR	Long-Wave Infrared
FPA	Focal Plane Array
SEM	Scanning Electron Microscopy
ROIC	Read-Out Integrated Circuit
AR	Anti-Reflection

## References

[1] Lawson W.D., Nielsen S., Putley E.H., Young A.S. (1959). Preparation and Properties of HgTe and Mixed Crystals of HgTe-CdTe. J. Phys. Chem. Solids.

[2] Rogalski A. (2005). HgCdTe Infrared Detector Material: History, Status and Outlook. Rep. Prog. Phys..

[3] Rogalski A., Antoszewski J., Faraone L. (2009). Third-Generation Infrared Photodetector Arrays. J. Appl. Phys..

[4] Kopytko M., Sobieski J., Gawron W., Martyniuk P. (2022). Study of HgCdTe (100) and HgCdTe (111)B Heterostructures Grown by MOCVD and Their Potential Application to APDs Operating in the IR Range up to 8 µm. Sensors.

[5] Madejczyk P., Manyk T., Rutkowski J. (2023). Research on Electro-Optical Characteristics of Infrared Detectors with HgCdTe Operating at Room Temperature. Sensors.

[6] Eich D., Ames C., Breiter R., Figgemeier H., Hanna S., Lutz H., Mahlein K.M., Schallenberg T., Sieck A., Wenisch J. (2019). MCT-Based High Performance Bispectral Detectors by AIM. J. Electron. Mater..

[7] Wang K., Yao C., Wu Y., Wang X., Wang Y., Li P. (2023). Laser-Interfered Studies in HgCdTe Infrared Focal Plane Array Detector by High-Repetition-Rate Mid-Infrared Supercontinuum Fiber Laser. Opt. Laser Technol..

[8] Tang W., Guo J., Shao J., Wang T. (2014). Analysis of Damage Threshold on HgCdTe Crystal Irradiated by Multi-pulsed CO_2_ laser. Opt. Laser Technol..

[9] Bartoli F., Kruer M., Esterowitz L., Allen R. (1973). Laser Damage in Triglycine Sulfate—Experimental Results and Thermal Analysis. J. Appl. Phys..

[10] Bartoli F., Esterowitz L., Kruer M., Allen R. (1975). Thermal Modeling of Laser Damage in 8–14 μm HgCdTe Photoconductive and PbSnTe Photovoltaic Detectors. J. Appl. Phys..

[11] Bartoli F., Esterowitz L., Allen R., Kruer M. (1976). A Generalized Thermal Model for Laser Damage in Infrared Detectors. J. Appl. Phys..

[12] Bartoli F., Esterowitz L., Kruer M., Allen R. (1975). Thermal Recovery Processes in Laser Irradiated HgCdTe (PC) detectors. Appl. Opt..

[13] Lei P., Li H., Bian J., Nie J. (2013). Experimental Study of HgCdTe Imaging Sensor Irradiated by TEA-CO_2_ Laser. Acta Opt. Sin..

[14] Zhang Y., Shao J., Tang W. (2021). Damage Effect of TEA CO_2_ Long Wave Infrared Laser on Detector Assembly of Infrared Staring Imaging System. Opt. Precis. Eng..

[15] Liu Y., Zhou F., Wang Y., Zhang Y., Zhang Y., Zheng H., Shao J. (2024). Experimental Study on Damage Effect of Mid-Infrared Pulsed Laser on Charge Coupled Device (CCD) and HgCdTe Detectors. Sensors.

[16] Zhang Y., Zheng C., Liu Y., Wang Y., Xu Y., Shao J. (2023). Damage Mechanism of HgCdTe Focal Plane Array Detector Irradiated Using Mid-Infrared Pulse Laser. Sensors.

[17] Chen C.S., Liu A.H., Sun G., He J.L., Wei X.Q., Liu M., Zhang Z.G., Man B.Y. (2006). Analysis of Laser Damage Threshold and Morphological Changes at the Surface of a HgCdTe Crystal. J. Opt. A Pure Appl. Opt..

[18] Li N., Li Z. (2025). The Enhancement Laser Dazzling Effect Induced by CW Laser Combined with Nanosecond Laser. Proc. SPIE.

[19] Ma Y., Zhou W., Chang H., Jian Z. (2024). Laser-Induced Interference to Infrared Detector Using Continuous Wave and Short-Pulse Lasers. Sensors.

[20] Gao Y., He H., Yang R., Yuan S., Wang X., Wang B., Jiang Y. (2025). Thermo-mechanical simulations of pulsed CO_2_ laser-HgCdTe detector. Infrared Phys. Technol..

